# Effect of cold atmospheric plasma treatment on the metabolites of human leukemia cells

**DOI:** 10.1186/s12935-019-0856-4

**Published:** 2019-05-17

**Authors:** Dehui Xu, Ning Ning, Yujing Xu, Bingchuan Wang, Qingjie Cui, Zhijie Liu, Xiaohua Wang, Dingxin Liu, Hailan Chen, Michael G. Kong

**Affiliations:** 1 State Key Laboratory of Electrical Insulation and Power Equipment, Centre for Plasma Biomedicine, Xi’an Jiaotong University, Xi’an, 710049 Shaanxi People’s Republic of China; 2 The School of Life Science and Technology, Xi’an Jiaotong University, Xi’an, 710049 Shaanxi People’s Republic of China; 3 Frank Reidy Center for Bioelectrics, Old Dominion University, Norfolk, VA 23508 USA; 4 Department of Electrical and Computer Engineering, Old Dominion University, Norfolk, VA 23529 USA

**Keywords:** Cold atmospheric plasma, Acute myeloid leukemia, Metabolite profiling, Alanine, aspartate and glutamate metabolism, d-Glutamine and d-glutamate metabolism

## Abstract

**Background:**

Acute myeloid leukemia (AML) is a typically fatal malignancy and new drug and treatment need to be developed for a better survival outcome. Cold atmospheric plasma (CAP) is a novel technology, which has been widely applied in biomedicine, especially in various of cancer treatment. However, the changes in cell metabolism after CAP treatment of leukemia cells have been rarely studied.

**Methods:**

In this study, we investigated the metabolite profiling of plasma treatment on leukemia cells based on Gas Chromatography Tandem Time-of-Flight Mass Spectrometry (GC-TOFMS). Simultaneously, we conducted a series of bioinformatics analysis of metabolites and metabolic pathways with significant differences after basic data analysis.

**Results:**

800 signals were detected by GC–TOF mass-spectrometry and then evaluated using PCA and OPLS-DA. All the differential metabolites were listed and the related metabolic pathways were analyzed by KEGG pathway. The results showed that alanine, aspartate and glutamate metabolism had a significant change after plasma treatment. Meanwhile, d-glutamine and d-glutamate metabolism were significantly changed by CAP. Glutaminase activity was decreased after plasma treatment, which might lead to glutamine accumulation and leukemia cells death.

**Conclusions:**

We found the above two metabolic pathways vulnerable to plasma treatment, which might result in leukemia cells death and might be the cornerstone of further exploration of plasma treatment targets.

**Electronic supplementary material:**

The online version of this article (10.1186/s12935-019-0856-4) contains supplementary material, which is available to authorized users.

## Background

Acute myeloid leukemia (AML) is a myeloid cancer, which is characterized by the rapid growth in the bone marrow and blood [1]. AML is mainly treated with long-term chemotherapy and radiation therapy for the purpose of induction of remission, or treated with stem cell transplantation [1, 2]. There is no doubt that these therapies bring about unavoidable harm to human normal cells. Therefore, it’s necessary to develop a new technology for the treatment of acute myeloid leukemia.

Cold atmospheric plasma (CAP) is a new technology that has attracted much attention in recent years especially in biomedical applications, such as bacterial disinfection, application of skin diseases, dentistry, cell transfection, wound healing and cancer treatment [3–8]. It is an ionized gas generated by electrical discharges in the atmospheric pressure at room temperature [9]. It has reported that plasma can effectively induce cell death in various types of cancer cells, including colon cancer, melanoma, cervical cancer, glioma, multiple myeloma and so on [10–17]. However, the effect of plasma treatment on the metabolites of tumor cell has been rarely reported. Cell metabolism, a general term for a series of ordered chemical reactions, is one of the most important physiological mechanism to maintain the normal growth and reproduction of organisms [18]. Cancer cells are able to achieve rapid and explosive proliferation due to metabolic reprogramming. Metabolic reprogramming is an oncogenic signaling, which facilitates assimilation of carbons into macromolecules such as lipids, proteins and nucleic acids to generate a large number of intermediate metabolites required for the growth and proliferation of cancer cells [19–22]. Therefore, it’s of great necessity to understand the effects of gas plasma on tumor cell metabolism, so as to treat cancer more precisely by plasma treatment. Based on the above notion, we performed a metabolomic analysis, which showed that the metabolites of leukemia cells have changed a lot after plasma treatment. Importantly, we found that alanine, aspartate and glutamate metabolism had a significant change, suggesting that alanine, aspartate and glutamate metabolism may exist critical targets for plasma treatment.

## Methods

### Surface plasma device

**Fig. 1:**
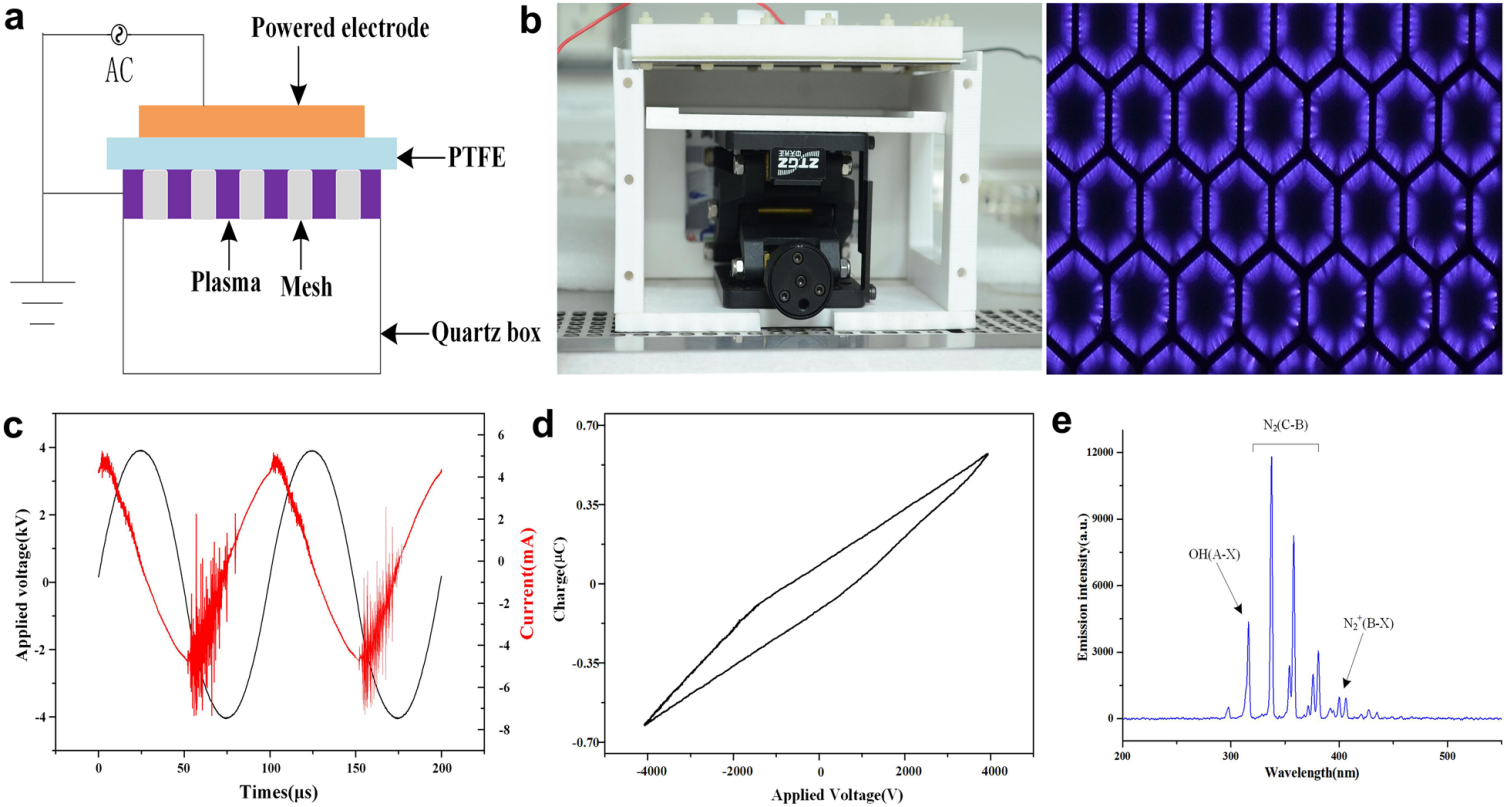
**a** Schematic diagram; **b** physical map and discharge photograph of the surface plasma; **c** discharge parameters; **d** V–Q Lissajous figure of the discharge powers; **e** emission spectra of the Surface plasma

In this study, we used a surface plasma device. As shown in Fig. 1a, the surface discharge structure of the plasma consisted of a high-voltage (HV) electrode, a ground electrode and a 1 mm thickness hexagonal polytetrafluroethylene (PTFE) sandwiched between the two electrodes. The surface plasma was generated when a sinusoidal high voltage at peak-to-peak value of 5 kV was applied. We used a HV probe (Tektronix, P6015A) and a current probe (Tektronix, P6021) to measure the discharge voltage and current respectively. Surface plasma was maintained at an electrical power of 0.06 W/cm^2^. The physical map of the surface discharge device and the plasma interface were shown in the Fig. 1b.

### Optical emission spectroscopy

We used a UV/visible spectrometer (Maya pro 2000, Ocean Optics, China) within a wavelength range of 200–800 nm to measure emission spectra of the surface plasma. The optical probe was mounted directly away the discharge area at 2 cm, which ensured a clear spectrum when detecting the spectrum.

### Cell culture condition

The MOLM13 leukemia cell line was used in this study. MOLM13 cells were cultured in Roswell Park Memorial Institute (RPMI) 1640 medium coupled with 10% fetal calf serum, 100 U/mL penicillin, and 50 µg/mL streptomycin (Gibco-Invitrogen, Carlsbad, CA, 15140-122). Cell culture bottles were placed at 37 °C in an incubator (Thermo Scientific, Waltham, MA, USA) containing 5% CO_2_. The medium was refreshed 24 h before performing experiments.

### Cell viability assessment

We used a Cell-Titer-Glo^®^ luminescent cell viability assay kit (Promega, Madison, WI, USA) to measure cell viability, which was based on quantitation of the ATP (Adenosine triphosphate) to determine the number of viable cells in culture medium. And we added 100 μL of samples and 100 μL of Cell-Titer-Glo^®^ reagent to the opaque-walled multiwell plate (96-well plates), and then the mixture was incubated at room temperature for 10 min. We utilized a microplate reader (Thermo Scientific Varioskan Flash, Waltham, MA, USA) with the protocol of “luminometric” measurement to detect the luminescence.

### Solvents and reagents

L-2-chlorophenylalanine was purchased from Hengbai Biotech Co Ltd (Shanghai, China). The methoxy amination hydrochloride (chromatographic grade), pyridine and chloroform were all from Admas (Shanghai, China). In addition, we bought BSTFA (including 1% TMCS, v/v) from REGIS Technologies Inc (Morton Grove, IL, USA) and methanol (HPLC grade) from ANPEL Laboratory Technologies Inc (Shanghai, China). Saturated fatty acid methyl lipids (C8, C9, C10, C12, C14, C16, C18, C20, C22, C24) were purchased from Dr. Ehrenstorfer (Augsburg, Germany). Deionized water (Thermo; Waltham, MA, USA) was used throughout the experiment. We used 20 µΜ/L and 40 µM/L BPTES in DMSO for subsequent experimental verification.

### Sample collection

We seeded 3 × 10^5^ cells/well in 300 μL of the medium in a 24-well plate. Wells were treated with gas plasma for 40 s as plasma treatment group, and the rest wells were control group without any treatment, containing 5 replicates/samples in each group. After incubation for 24 h, cells were collected and counted to ensure that the number of cells was about 1 × 10^7^ cells/sample. Cells were centrifuged at 4 °C for 5 min at the speed of 135*g* and washed 3 times at 4 °C with PBS at the speed of 76*g*. Then the cell mass in EP tube was placed in liquid nitrogen for 5 min rapidly and stored in the − 80 °C refrigerator until it was analyzed.

### Sample preparation

Before metabolite analysis, sample was that 0.6 ml extract (V methanol: chloroform = 3:1) in 2 ml EP tube and 10 μL L-2-cholrophenylalanine (1 mg/mL stock in dH_2_O) as internal standard were mixed. After vortex mixing for 30 s, steel balls were added and ground at 45 Hz for 4 min, and then sonicated in ice water for 5 min. Next, the above step was repeated 3 times. After centrifugation at 15,871*g*, 4 °C for 15 min, the supernatant (0.5 mL) was transferred to a fresh 2 ml GC/MS glass vial. Then, the extracted metabolites were dried in a vacuum concentrator without heating, and 30 μL of methoxylamine hydrochloride was added. After incubation in oven at 80 °C for 30 min, 40 μL of BSTFA reagent (1% TMCS, v/v) was thoroughly mixed with the sample aliquots and incubated at 70 °C for 1.5 h to obtain a derivative metabolite for GC–MS analysis.

### GC–TOF–MS analysis

We performed GC–TOF–MS analysis using an Agilent 7890 gas chromatograph system coupled with a Pegasus HT Time-of-Flight Mass Spectrometer. The system utilized a DB-5MS capillary column coated with 5% diphenyl cross-linked with 95% dimethylpolysiloxane (30 m × 250 μm inner diameter, 0.25 μm film thickness; J&W Scientific, Folsom, CA, USA). 1 μL aliquot of the analyte was injected in splitless mode. Helium was used as the carrier gas, the front inlet purge flow was 3 mL min^−1^, and the gas flow rate through the column was 1 mL min^−1^. The initial temperature was kept at 50 °C for 1 min, then raised to 310 °C at a rate of 10 °C min^−1^, then kept for 8 min at 310 °C. The injection, transfer line, and ion source temperatures were 280, 280, and 250 °C, respectively. The energy was − 70 eV in electron impact mode. The mass spectrometry data were acquired in full-scan mode with the m/z range of 50–500 at a rate of 20 spectra per second after a solvent delay of 6.27 min.

### Data preprocessing and annotation

We used Chroma TOF 4.3X software of LECO Corporation and LECO-Fiehn Rtx5 database for exacting raw peaks, filtering the data baselines and calibration of the baseline, peak alignment, deconvolution analysis, peak identification and integration of the peak area [23]. Both of mass spectrum match and retention index match were considered in metabolites identification.

### Spectrophotometric detetion of GLS activity

We used a glutaminase (GLS) activity assay kit (Comin, Suzhou, China) to measure GLS activity. The principle was to measure the rate of ammonia production from glutamine catalyzed by GLS to calculate the enzymatic activity. We first collected leukemia cells into a centrifuge tube (1 million cells), washed them 2–3 times with PBS, centrifuged and discard the supernatant, and then added 400 µL extract, ultrasonically disrupted cells (sonicate for 7–8 s, interval for 10–15 s, repeat 10 times, ice bath) with power 200 W. After the above steps were completed, we centrifuged the disrupted cells for 10 min at 4 °C to take the supernatant. The supernatant and the reagent 1, 2 were mixed in 37 °C water bath for 1 h. Last, we used a spectrophotometer to read the absorbance of ammonia at 420 nm.

## Results

### Plasma discharging parameter and characters

A sinusoidal power supply at *f *= 10 kHz and Vpp = 5 kV was used to generate a surface plasma in ambient air. Figure 1c showed the applied AC voltage curve and the corresponding current curve when the surface plasma discharging. A V–Q Lissajous figure of the discharge powers was shown in Fig. 1d. The discharging characteristic of the surface plasma was depicted by an emission spectra, as shown in Fig. 1e. There were several spectral lines in the surface plasma (e.g. OH (A) 310 nm, N_2_ (C) 340 nm, N_2_^+^ (B) 390 nm).

### Metabolic profiles of plasma-treated cells by GC–TOF

**Fig. 2:**
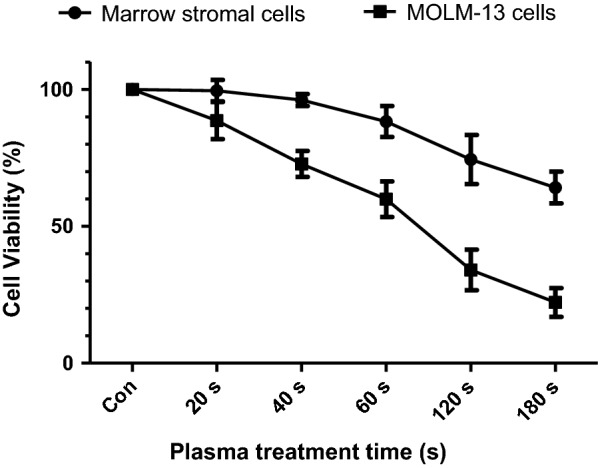
Cell viability of normal bone marrow stromal cells and MOLM-13 cells by plasma treatment

**Fig. 3:**
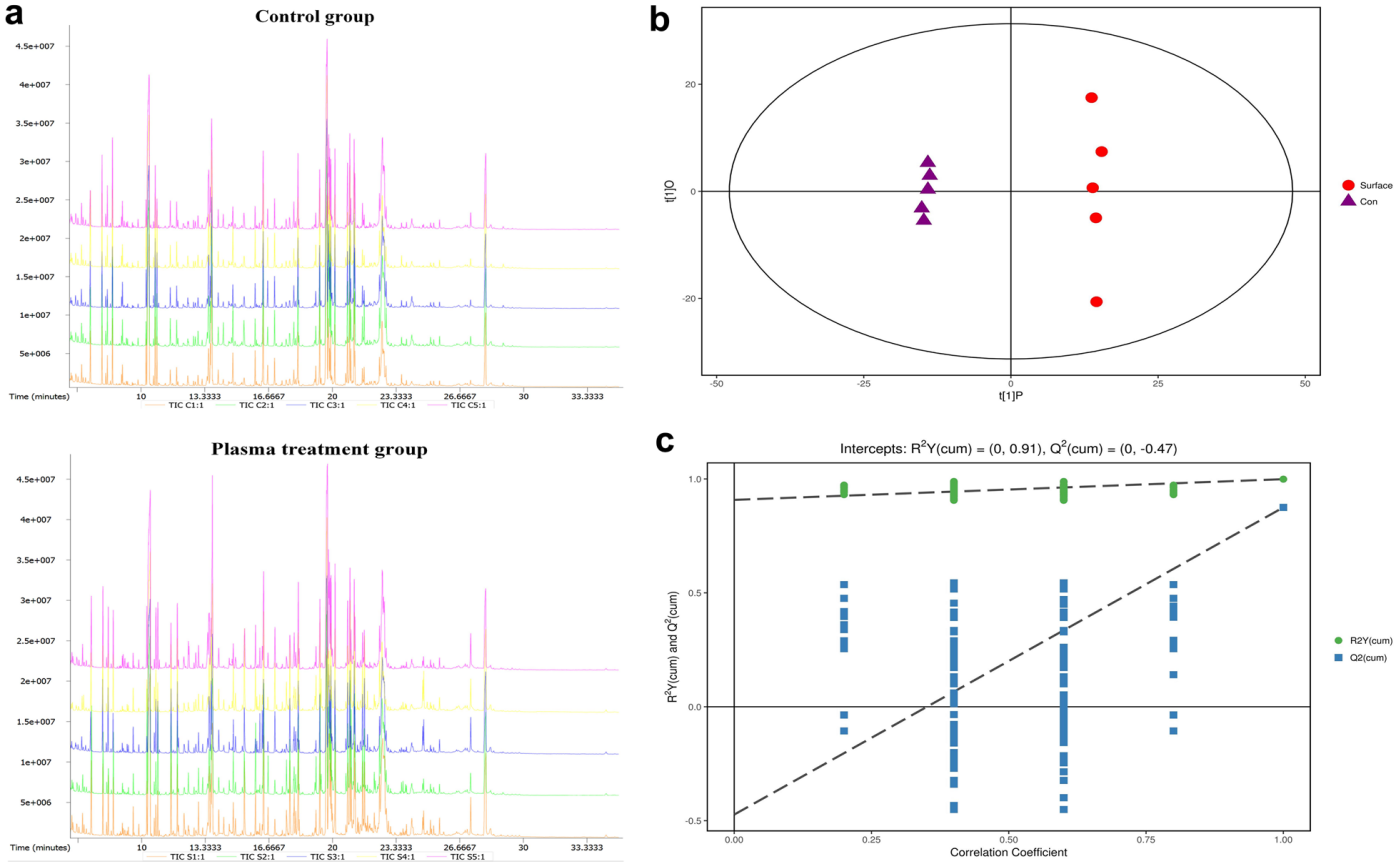
**a** GC-TOFMS total ion chromatogram; **b** score scatter plot of OPLS-DA model; **c** permutation test of OPLS-DA model

We totally investigated 10 samples of MOLM13 leukemia cell line, of which five samples as the experimental group were treated with plasma for 40 s and the other five samples were not treated as experimental controls. Figure 2 showed that cell viability of MOLM13 cell line was decreased significantly with increasing plasma treatment time while cell viability of normal cell line derived from normal bone marrow stromal cells was decreased slightly. And the leukemia cell activity after the plasma treatment for 40 s is about 70%, which is especially conducive to further metabolic analysis. Using Gas Chromatography Tandem Time-of-Flight Mass Spectrometry (GC-TOFMS), we extracted 837 signals based on mass spectral deconvolution software for peak detection. In order to better analyze the data, we performed a series of preprocessing on the raw data and finally 800 signals were retained. Figure 3a showed the GC-TOFMS total ion chromatogram of plasma treatment group and control group. The database mapping of metabolites was listed in Additional file 1.

### Unsupervised and supervised evaluation of metabolite signatures

First, we made a principal component analysis (PCA) after preprocessing the raw data. Because variables contain both differential variables related to categorical variables and large quantities of them irrelevant to each other, the result of metabolic differences was not very significant, which was basically in the 95% confidence interval (Hotelling’s T-squared ellipse). Therefore, this data was be further analyzed using orthogonal least squares discriminant analysis (orthogonal projection to latent structure discriminant analysis, OPLS-DA). Different from PCA, OPLS-DA was a supervised statistical method for discriminant analysis. This method used OPLS-DA to establish a relationship model between metabolite expression levels and sample categories to achieve prediction of sample categories. We filtered out the orthogonal variables that were independent of the classified variables in the metabolites and analyzed the differences between non orthogonal and orthogonal variables in order to obtain more reliable metabolites. The parameters of the OPLS-DA model were shown in the statistical model parameter Table 1. R^2^X and R^2^Y represented the interpretation rates of the model for the matrix X and Y respectively, and Q^2^ denoted the prediction ability of the model. Theoretically, the closer the value of R^2^ and Q^2^ is to 1, the better the model is Table 1 showed that our OPLS-DA model could be used for further verification.

**Table 1:** Statistical model parameters table of OPLS-DA model

Model	Type	A	N	R^2^X (cum)	R^2^Y (cum)	Q^2^ (cum)
Model 1	OPLS-DA	1 + 1 + 0	10	0.426	0.999	0.875

As Fig. 3b shows, the X-axis denoted the predicted principal component score of the first principal component, while the Y-axis showed the orthogonal principal component score. The different scatter shape and color represented different experimental groups respectively. The results of the OPLS-DA score plot showed that the two groups of samples were very distinct, and the samples were all at the 95% confidence interval. The OPLS-DA permutation test was to avoid overfitting of the test model and to ensure statistical significance of the evaluation model. The result of the permutation test of the OPLS-DA model between plasma treatment group and control group was shown in the Fig. 3c. The X-axis and Y-axis represented the degree of substitution retention of the permutation test and the value of R^2^Y or Q respectively. Green circles and blue square points denoted R^2^Y value and Q value, which were obtained by the permutation test respectively. The two dotted lines indicated the R^2^Y’s and Q’s regression lines. The result showed that the original model had good robustness and there was no overfitting phenomenon.

### Screening of differential metabolites

**Fig. 4:**
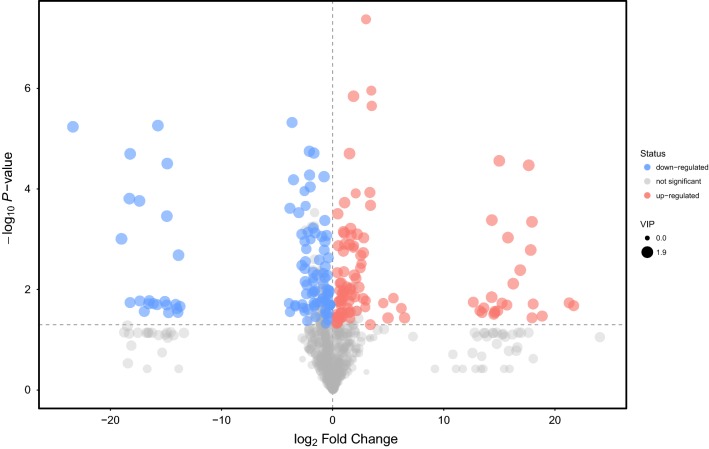
Volcano plot of differential metabolites in plasma treatment group and control group. Red represented up-regulated metabolites; Blue represented down-regulated metabolites; Gray represented metabolites that have no significant change

Based on the inherent characteristics of GC-TOFMS metabolomic data, we used multivariate statistical analysis methods to analyze the data and screened out differential metabolites (Additional file 2) between plasma treatment group and control group. *P* value of student’s t-test is less than 0.05 and the first principal component’s Variable Importance in the Projection (VIP) is greater than 1. Volcano plot was a kind of image used to show the difference data between groups, where the X-axis represented the fold change of the plasma treatment group compared to the control group (base 2 logarithm) and the Y-axis represented the student’s t-test P-value (base 10 logarithm). We visualized the above results of screening differential metabolites in the form of volcano plot (Fig. 4). The result showed the significantly up-regulated metabolites (red), down-regulated metabolites (blue), and non-significant differential metabolites (gray). The scatter size represented the VIP value of the OPLS-DA model. The larger scatter was on behalf of the bigger VIP value.

### Cluster analysis

Heatmap uses color changes to reflect data information in a two-dimensional matrix or table. It can visually represent the size of data value with defined depths of color. The hierarchical clustering analysis can clear classify the metabolites with the same and different characteristics between the sample groups. The clustered data are represented on the heatmap, and the high abundance and low abundance species can be clustered. The similarity and diversity of the community composition at different levels can be reflected by the color gradient and similarity.

**Fig. 5:**
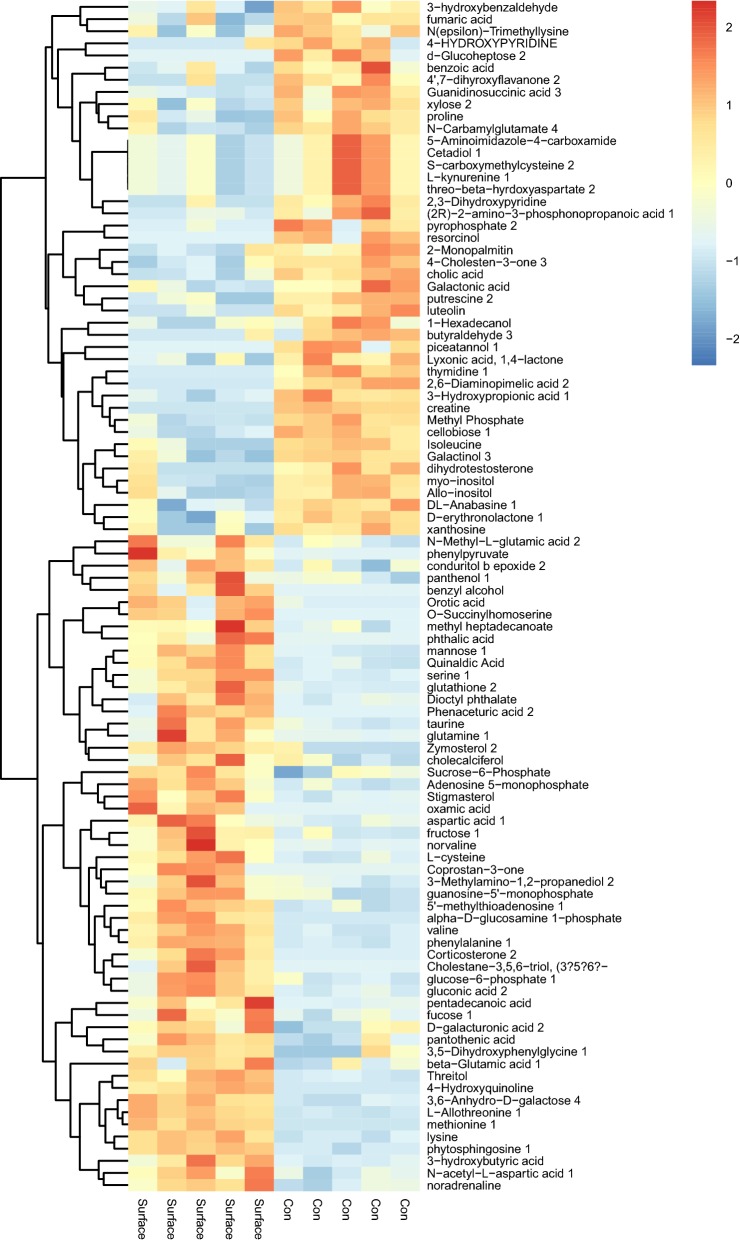
A heatmap was drawn to show the differential expressed metabolites. Up-regulated expressed metabolites were shown in red; Down-regulated expressed metabolites were shown in blue. *P < 0.05

After hierarchical clustering analysis of the differential metabolites between the surface plasma treatment group and the control group, we visualized the obtained results in a heatmap (Fig. 5). Clustering of samples using the significantly regulated metabolites resulted in a nearly perfect separation of the plasma treatment group and the control group. It indicated that there were significant differences in the expression of metabolites between the two groups.

### All the pathways relevant to differential metabolites by KEGG analysis

All the metabolites do not work alone and they are involved with one or more metabolic pathways together with other metabolites. Deregulation of differential metabolites is also the result of mutual influence, which even changes the expression of their own metabolic pathways.

**Fig. 6:**
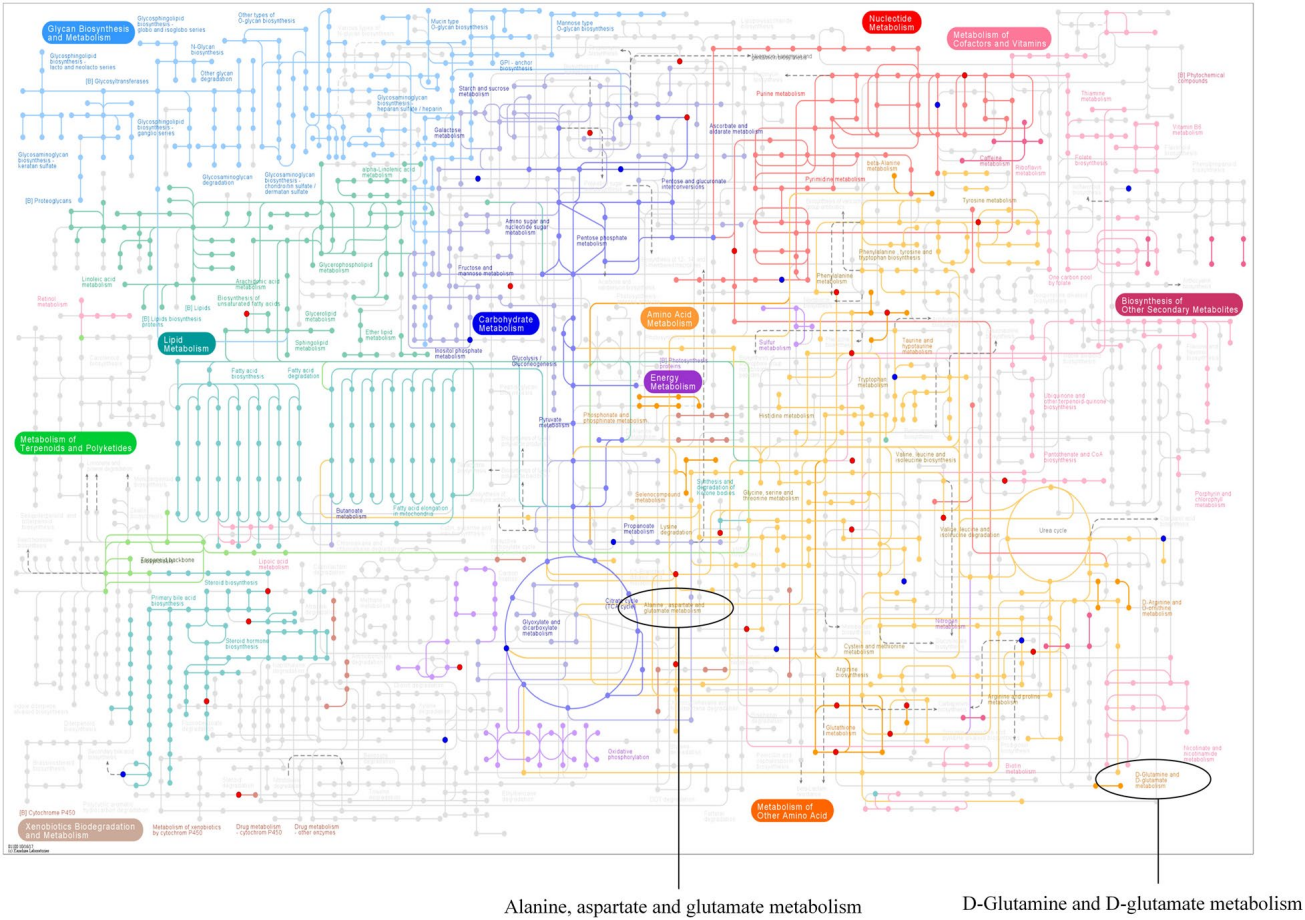
KEGG pathway map with bright red/blue dots representing the differentially expressed metabolites. Bright red dots represented up-regulated metabolites; Bright blue dots represented down-regulated metabolites

KEGG (Kyoto Encyclopedia of Genes and Genomes) is a huge database used to systematically analyze gene functions, which can link genomic information to metabolites functional information [24]. The PATHWAY database utilizes a few direct homologous tables to obtain information about conserved subpathways that is usually encoded by positionally coupled genes on the chromosome, which is particular useful for further understanding the metabolic changes of the pathway [24]. We mapped all 800 metabolites to Homo sapiens in the KEGG pathway database. And we also listed all the pathways for mapping differential metabolites, as shown in Additional file 3. Next, we marked the differential metabolites on the KEGG pathway map. As shown in Fig. 6, bright red dots represented up-regulation, while bright blue dots represented down-regulation.

### Metabolic pathway analysis related with differential metabolites

To know whether these pathways were significantly affected after plasma treatment, KEGG analysis was not enough, therefore we further analyzed metabolic pathways for differential metabolites. By comprehensive analysis of pathways where differential metabolites were located (including enrichment analysis and topological analysis), we further screened pathways and found the key pathways with the highest correlation with differential metabolites. Metabolic pathway analysis results were shown in Additional file 4.

**Fig. 7:**
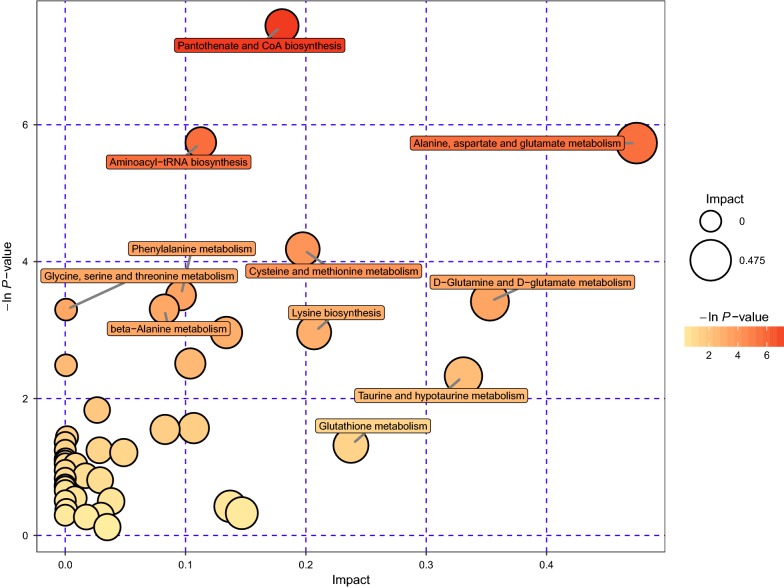
Bubble diagram of differential metabolic pathways. One bubble represented one metabolic pathway; Differentially expressed metabolic pathways have been labeled

The results of the metabolic pathway analysis were shown as bubble diagram (Fig. 7). One metabolic pathway was represented by one bubble in the diagram. The location of the X-axis of a bubble and the size of the bubble indicated impact value of a metabolic pathway, which was from the topological analysis of metabolic pathway. The location of the Y-axis of a bubble and the color of the bubble indicated the P value of the enrichment analysis. Pathways with significant metabolic differences was screened by considering impact value of the topological analysis and P value of the enrichment analysis. From the bubble diagram, we could find out that alanine, aspartate and glutamate metabolism pathway was the most significant changes after gas plasma treatment in MOLM13 cells. Furthermore, it was worth noting that d-glutamine and d-glutamate metabolism were also significantly changed in leukemia cells.

### Inhibition of GLS after plasma treatment leading to disruption of glutamine metabolism

We found that glutamine in alanine, aspartate and glutamate metabolism pathway and in d-glutamine and d-glutamate metabolism pathway was up-regulated in plasma treatment group (Fig. 5). Studies have shown that glutamine metabolism plays an important role in biosynthesis, energy metabolism and cell homeostasis of tumor cells and promotes tumor growth [25, 26]. Moreover glutaminase (GLS) is overexpressed in many tumor cells and converts glutamine to glutamic acid, which is then converted to $\propto$-KG and introduced into TCA cycle [25]. We therefore hypothesized that glutaminase activity of plasma-treated leukemia cells was reduced and glutamine could not be normally metabolized and converted to glutamic acid, which suppressed the proliferation of leukemia cells and even leaded to leukemia cells apoptosis. This also explained why alanine, aspartate and glutamate metabolism are abnormal after plasma treatment.

**Fig. 8:**
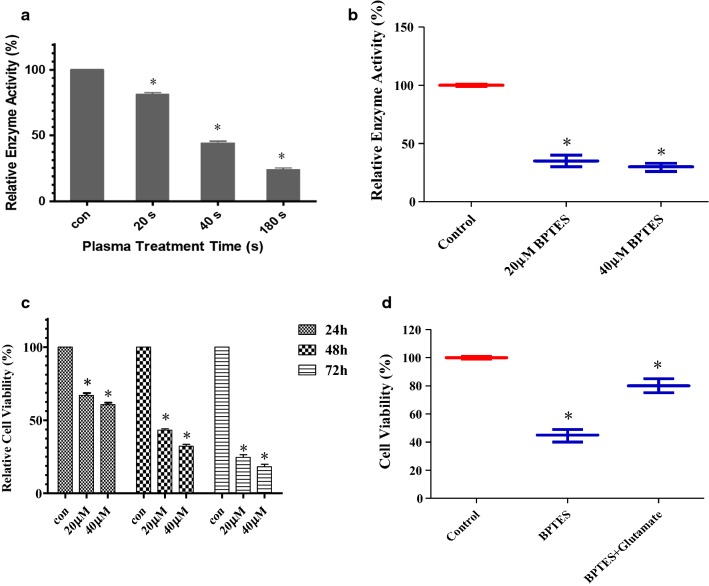
Relative glutaminase activity of **a** leukemia cells after plasma treatment and **b** leukemia cells with 20 µM/L and 40 µM/L BPTES; Relative cell viability of **c** leukemia cells with 20 µM/L and 40 µM/L BPTES and **d** leukemia cells with 20 µM/L BPTES vs. 20 µM/L BPTES adding glutamate. *P < 0.05

To determine whether the differentially metabolic pathway and the differential metabolite were responsible for leukemia cells death, we analyzed glutaminase activity of leukemia cells before and after plasma treatment. The result showed that glutaminase activity after plasma treatment was reduced (Fig. 8a). Subsequently, we inhibited glutaminase activity with 20 µM/L and 40 µM/L BPTES (bis-2-(5-Phenylacetmido-1,2,4-Thiadiazol-2-yl)Ethyl Sulfide, GLS inhibitor) for 24 h, 48 h and 72 h. We found that when glutaminase was inhibited (Fig. 8b), leukemia cells activity was decreased (Fig. 8c). Interestingly, when we added glutamate to the experimental group containing 20 µM/L BPTES for 48 h, relative cell activity had a certain increase (Fig. 8d).

## Discussion

As a new developed technology, atmospheric-pressure-cold plasma has aroused widespread concern in biomedical applications. It has reported that atmospheric cold plasma can selectively induce various tumor cells death [27, 28]. And a large number of metabolites have been shown to contribute to distinguish tumors from healthy tissue [29]. Therefore, it’s a new perspective to explore changes in metabolites and metabolic pathways of tumor cells before and after plasma treatment. This metabolic study might be useful to identify metabolic pathways that could be targeted for plasma treatment. In this way, the bioenergetic state of the tumor can be destroyed more specifically. In our study, we investigated the changes in cell metabolism after CAP treatment of leukemia cells by GC–TOF–MS analysis. From results, we found that significant differences in metabolites between plasma treatment group and control group. By multivariate analysis, we screened for differential metabolites that were significantly up-regulated or down-regulated. The changes in the level of these differential metabolites were not independent. On the contrary, they had mutual promotion or antagonism among them, which might affect the level of certain metabolic pathways and further affect the viability and metabolic level of cells. Therefore, it’s important to analyze the metabolic pathways that have the highest correlation with differential metabolites. It has reported that several drugs such as A1CAR and A-76969662 were able to upregulate AMPK signaling directly or indirectly by activating the AMPK protein complex so as to inhibit leukemia cells growth and even induce apoptosis [30–32]. However, by KEGG analysis of the metabolic pathways, we found that alanine, aspartate and glutamate metabolism had significant change while AMPK signaling pathway had no change after plasma treatment in leukemia MOLM13 cells. We next focused on glutamine, the differential metabolite in alanine, aspartate and glutamate metabolism pathway and in d-glutamine and d-glutamate metabolism pathway, because many studies have showed that glutamine plays an important role in signal transduction and proliferation of tumor cells [26, 33]. The first step of glutamine catabolism occurs through the activation of glutaminase (GLS), which catalyzes the conversion of glutamine to glutamic acid. Inhibition of glutaminase can inhibit leukemia cell growth and even induce apoptosis [34]. In cluster analysis, we found that glutamine was upregulated after plasma treatment. In order to determine its reason, we investigated that GLS activity in leukemia cells after plasma treatment, and the result showed that GLS activity was decreased. We also inhibited GLS activity of leukemia cells by BPTES and found that inhibiton of GLS activity reduced cell viability. However, when added glutamate to leukemia cells inhibited GLS activity, we found increased relative cell activity. The above results showed that CAP treatment could inhibit the GLS activity of leukemia cells so that glutamine was not able to be normally metabolized to produce glutamic acid and thus accumulated, which might lead to leukemia cells death due to the lack of required nutrients. Our current work initially screened metabolites and metabolic pathways with significant differences of leukemia cell after CAP treatment with reduction of viability. At the same time, we used pathway inhibitors to manipulate the perturbed key pathway and analyzed the causes and effects of this pathway change. With more details about the changes of metabolic pathways induced by CAP treatment, it will be a breakthrough to improve the treatment effect by CAP in tumor therapy of leukemia or even other tumors.

## Conclusions

In conclusion, we analyzed the differential metabolites in leukemia cell between plasma treatment group and control group by bioinformatics analysis. More importantly, we found a crucial differential metabolic pathway, alanine, aspartate and glutamate metabolism pathway, which was vulnerable to plasma treatment. Its changes may lead to leukemia cells apoptosis. Metabolomic analysis is therefore a promising approach to investigate the key targets of plasma-treated tumor. The present study may be a meaningful finding for further screening the optimum target of plasma treatment for tumors.

## Additional files

**Additional file 1: Table S1.** Metabolite mapping.

**Additional file 2: Table S2.** Differential metabolites.

**Additional file 3: Table S3.** KEGG pathway.

**Additional file 4: Table S4.** Pathway analysis.

## Data Availability

The datasets generated during and/or analyzed during the current study are available from the corresponding author on reasonable request.

## Abbreviations

AML: acute myeloid leukemia
GC–TOF: gas-chromatography time-of-flight
CAP: cold atmospheric plasma
PCA: principal component analysis
VIP: variable importance in the projection
KEGG: Kyoto Encyclopedia of Genes and Genomes
ATP: adenosine triphosphate
CoA: coenzyme A
GLS: glutaminase
BPTES: bis-2-(5-Phenylacetmido-1,2,4-Thiadiazol-2-yl)Ethyl Sulfide
